# Retinal Pigment Epithelium Cell Density and Bruch’s Membrane Thickness in Secondary versus Primary High Myopia and Emmetropia

**DOI:** 10.1038/s41598-020-62096-7

**Published:** 2020-03-20

**Authors:** Jost B. Jonas, Dong Li, Leonard Holbach, Songhomitra Panda-Jonas

**Affiliations:** 1 Department of Ophthalmology, Medical Faculty Mannheim, Heidelberg University, Mannheim, Germany; 2 Beijing Tongren Eye Center, Beijing Key Laboratory of Intraocular Tumor Diagnosis and Treatment, Beijing Ophthalmology & Visual Sciences Key Lab, Beijing Tongren Hospital, Capital Medical University, Beijing, China; 3 Department of Ophthalmology, Friedrich-Alexander University Erlangen-Nürnberg, Erlangen, Germany; 4 Augenpraxis Jonas, Heidelberg, Germany

**Keywords:** Neuroscience, Anatomy

## Abstract

To assess differences between secondary high myopia (SHM) due to congenital glaucoma and primary high myopia (PHM) and non-highly myopic eyes (NHM) in the relationships between axial length and Bruch’s membrane (BM) thickness and retinal pigment epithelium (RPE) density. The histomorphometric study included human globes enucleated for reasons such as malignant uveal melanoma, end-stage painful secondary angle-closure glaucoma and congenital glaucoma. BM thickness and RPE cell density were measured upon light microscopy. The investigation included 122 eyes (mean axial length: 26.7 ± 3.7 mm; range: 20.0–37.0 mm): 7 eyes with SHM (axial length: 33.7 ± 2.1 mm; range: 31.0–37.0 mm), 56 eyes with PHM (mean axial length: 29.1 ± 2.4 mm; range: 26.0–36.0 mm) and 59 eyes in the NHM-group (axial length: 23.5 ± 1.3 mm; range: 20.0–25.5 mm). In the SHM group, longer axial length was associated with lower RPE cell density at the posterior pole (standardized regression coefficient beta: 0.92; non- standardized regression coefficient B: −2.76; 95% confidence interval (CI): −4.41, −1.10;*P* = 0.01), at the midpoint posterior pole/equator (beta: −0.87; B: −3.60; 95% CI: −6.48, −0.73;*P* = 0.03), and at the equator (beta: −0.88; B: −0.95; 95% CI: −1.68, −0.23; *P* = 0.02), but not at the ora serrata (*P* = 0.88). In the PHM-group and NHM group, RPE cell density at the posterior pole (*P* = 0.08) and ora serrata (*P* = 0.88) was statistically independent of axial length, while at the midpoint posterior pole/equator (*P* = 0.01) and equator (*P* < 0.001), RPE cell density decreased with longer axis. BM thickness in the SHM group decreased with longer axial length at the posterior pole (beta: −0.93;B: −0.29; 95% CI: −0.39, −0.14; *P* = 0.003), midpoint posterior pole/equator (beta: −0.79; B: −0.22; 95% CI: −0.42, −0.02; *P* = 0.035) and equator (beta: −0.84; B: −0.21; 95% CI: −0.37, −0.06; *P* = 0.017), while in the PHM-group and NHM-group, BM thickness at any ocular region was not statistically significantly correlated with axial length (all *P* > 0.05). In the SHM-group, but not in the PHM-group or NHM-group (*P* = 0.98), lower BM thickness was associated with lower RPE cell density (beta: 0.93; B: 0.09; 95% CI: 0.04, 0.14; *P* = 0.007), while in the eyes without congenital glaucoma the relationship was not statistically significant. In SHM in contrast to PHM, BM thickness and RPE cell density decrease in a parallel manner with longer axial length. The findings fit with the notion of BM being a primary driver in the process of axial elongation in PHM as compared to SHM.

## Introduction

Previous studies have shown that the thickness of the choroid and sclera decrease with longer axial length, more marked at the posterior pole and least marked in the retro-equatorial region^1–4^. Recent investigations suggested that the thickness of Bruch’s membrane, in contrast to scleral thickness and total choroidal thickness, was not correlated with axial length: Axially elongated eyes and eyes with a normal axial length did not differ in BM thickness^5,6^. It was assumed that the process of axial elongation took place primarily in the equatorial region by new production and enlargement of BM in that region, so that the posterior pole was pushed backward and the globe assumed an axially elongated shape^7^. Since in that model the enlargement of BM occurred in a circumscribed region in the fundus periphery, BM thickness and subsequently the thickness of the retina and the density of the retinal pigment epithelium (RPE) at the posterior pole were not primarily affected by that process^7–9^.

While in that model of primary myopia the globe enlarged primarily in the equatorial region, the globe enlargement in eyes with secondary high myopia due to congenital glaucoma occurs mostly in all regions of the eye, including the cornea and pars plana region^10^. If the notion of BM enlargement occurring selectively in the equatorial region in eyes with primary myopia validly explains the independence of BM thickness and of RPE cell density at the posterior pole from axial length, eyes with secondary high myopia due to congenital glaucoma should have a reduced BM thickness and a reduced RPE cell density at the posterior pole. We therefore conducted this study to examine whether in eyes with secondary high myopia BM thickness and the RPE cell density decrease with longer axial length. The results could be of interest to further elucidate the process of emmetropization and myopization.

## Methods

The study population consisted of enucleated eyes of patients of European descent. The diagnosis of a malignant choroidal melanoma and or end-stage painful glaucoma were causes for the removal of the globes. The study was performed according to the Declaration of Helsinki guidelines. The Medical Ethics Committee II of the Medical Faculty Mannheim of the Heidelberg University approved the study. The Medical Ethics Committee waived the necessity of an informed consent by the patients, since the eyes had been removed up to 50 years before the start of the study. Most of the eyes with congenital glaucoma and eyes without congenital glaucoma had already previously been examined and included in a previous study on BM thickness and other histomorphometric investigations^2,5,7–9,11^. For the present investigation, all eyes were re-measured in a masked manner by an examiner experienced in the histomorphometry of the eye (JBJ).

After the eyes had surgically been removed, they were immediately fixed in a solution of 1% glutaraldehyde and 4% formaldehyde. The globes were kept in that solution for seven days at room temperature. The sagittal, vertical and horizontal globe diameter were subsequently measured. A central segment with a thickness of about 8 mm and running through the optic nerve head and the pupil was cut out of the fixed globes, dehydrated in alcohol, imbedded it in paraffin, sectioned for light microscopy, and stained by the Periodic-Acid-Shiff method or with hematoxylin eosin. Its meridional orientation depended on the location of the tumor in the group of eyes with a malignant choroidal melanoma, while the direction of the segment was horizontal in all other eyes. For the further examination, we used one section per globe. It had a thickness of 4–6 µm and ran through the central part of the pupil and optic nerve head. Using the in-built millimeter scale in the objective of the microscope, we measured histomorphometrically the thickness of BM and the density of the RPE cells as cell count per 300 μm. We defined high myopia by an axial length of ≥26.0 mm.

Using a statistical software program (SPSS for Windows, version 25.0; IBM-SPSS, Chicago, Illinois, USA), we assessed the mean values, standard deviations and 95% confidence intervals (CI) of the main outcome parameters (e.g., RPE cell density, BM thickness). We differentiated between eyes with secondary high myopia due to congenital glaucoma (SHM-group), eyes with primary high myopia (without congenital glaucoma) (PHM-group) and non-highly myopic eyes (NHM-group). Applying the student t-test or the Mann-Whitney test for unpaired samples, we determined the significance of differences in these parameters between the various groups. We calculated linear regression analyses between the outcome parameters and axial length, and determined the standardized regression coefficient beta, the non-standardized regression coefficient B, and its 95% confidence intervals (CI). The level of significance was 0.05 (two-sided) in all statistical tests.

## Results

The investigation included 122 eyes (mean axial length: 26.7 ± 3.7 mm; range: 20.0–37.0 mm) of 122 patients (mean age: 61.1 ± 16.4 years; range: 24–89 years). The study population included 7 eyes of 7 patients with secondary high myopia due to congenital glaucoma (SHM-group) (axial length: 33.7 ± 2.1 mm; range: 31.0–37.0 mm), 56 eyes with primary high myopia (without congenital glaucoma) (PHM group) (mean axial length: 29.1 ± 2.4 mm; range: 26.0–36.0 mm), and 59 non-highly myopic eyes (NHM group) (mean axial length: 23.5 ± 1.3 mm; range: 20.0–25.5 mm). Within the PHM-group, 42 (75%) eyes had glaucoma, and 20 (34%) eyes of the NHM-group had glaucoma.

**Figure 1:**
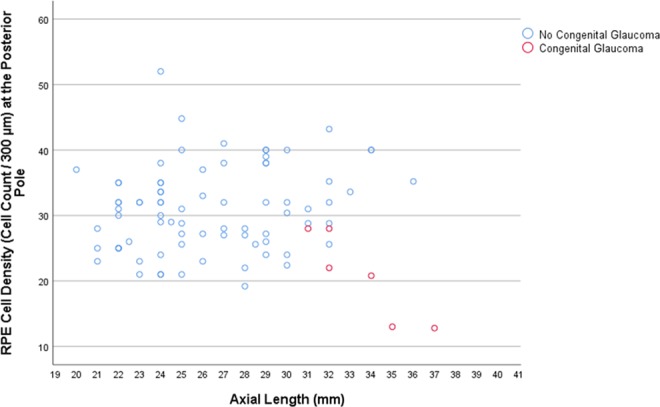
Scatterplot showing the association between axial length and retinal pigment epithelium (RPE) cell density at the posterior pole in eyes with congenital glaucoma versus eyes without congenital glaucoma.

**Figure 2:**
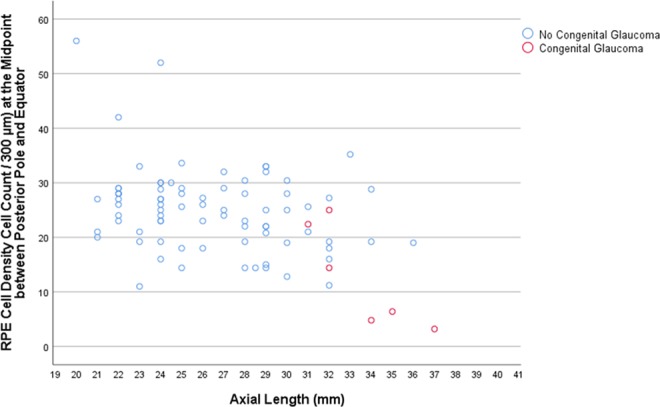
Scatterplot showing the association between axial length and retinal pigment epithelium (RPE) cell density at the midpoint posterior pole/equator in eyes with congenital glaucoma versus eyes without congenital glaucoma.

**Figure 3:**
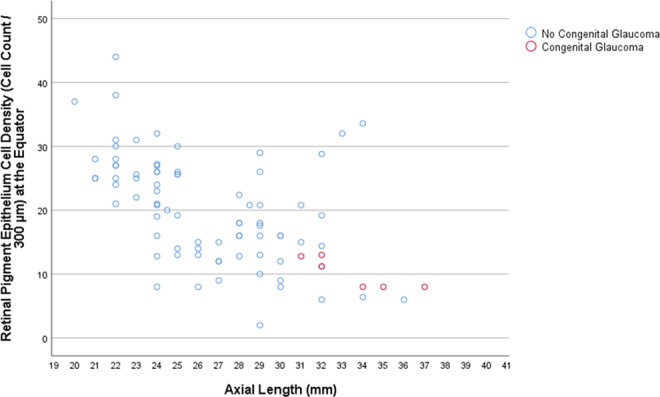
Scatterplot showing the association between axial length and retinal pigment epithelium (RPE) cell density at the equator in eyes with congenital glaucoma versus eyes without congenital glaucoma.

In the SHM group, longer axial length was associated with a lower mean RPE cell density measured at the posterior pole (beta: 0.92; B: −2.76; 95% CI: −4.41, −1.10; *P* = 0.01), at the midpoint between posterior pole and equator (beta: −0.87; B: −3.60; 95% CI: −6.48, −0.73; *P* = 0.03), and at the equator (beta: −0.88; B: −0.95; 95% CI: −1.68, −0.23; *P* = 0.02), while the association for the RPE cell density measured close to the ora serrata was not statistically significant (*P* = 0.88) (Table 1) (Figs. 1–3). In the eyes without congenital glaucoma, the RPE cell density at the posterior pole (*P* = 0.08) and close to the ora serrata (*P* = 0.88) was statistically independent of axial length, while at the midpoint posterior pole/equator (*P* = 0.01) and at the equator (*P* < 0.001), the RPE cell density decreased with longer axis (Table 1) (Figs. 1–3).

**Table 1:** Results of the linear regression analysis of the association between the density of retinal pigment epithelium (RPE) cells (cell count/300 µm) and axial length.

Region	RPE cell density (cells/300 µm)	Standardized Regression Coefficient beta	Non- Standardized Regression Coefficient B	95% Confidence Interval of B	*P*-Value*
**Secondary High Myopia (Congenital Glaucoma)**					
Posterior pole	20.8 ± 6.8	0.92	−2.76	−4.41, −1.10	0.01
Midpoint posterior pole and equator	21.4 ± 6.8	−0.87	−3.60	−6.48, −0.73	0.03
Equator	10.2 ± 2.5	−0.88	−0.95	−1.68, −0.23	0.02
Pre-Ora serrata	19.0 ± 6.0	0.10	0.36	−6.32, 7.03	0.88
**No Secondary High Myopia (No Congenital Glaucoma)**					
Posterior pole	31.0 ± 6.5	0.20	0.35	−0.04, 0.75	0.08
Midpoint posterior pole and equator	25.0 ± 7.7	−0.29	−0.59	−1.05, −0.14	0.01
Equator	20.2 ± 8.4	−0.50	−1.14	−1.59, −0.70	<0.001
Pre-Ora serrata	25.0 ± 7.1	−0.01	−0.03	−0.48, 0.43	0.91
**Primary High Myopia**					
Posterior pole	31.7 ± 6.4	0.26	0.70	−0.15, 1.55	0.10
Midpoint posterior pole and equator	23.2 ± 6.2	−0.14	−0.36	−1.21, 0.49	0.40
Equator	15.7 ± 7.1	0.14	0.41	−0.56, 1.38	0.40
Pre-Ora serrata	24.5 ± 7.8	0.19	0.64	−0.44, 1.72	0.24
**Non-Highly Myopic Group**					
Posterior pole	30.3 ± 6.6	0.14	0.67	−0.93, 2.28	0.40
Midpoint posterior pole and equator	26.7 ± 8.6	−0.22	−1.40	−3.49, 0.68	0.18
Equator	24.9 ± 6.9	−0.49	−2.48	−3.94, −1.02	0.001
Pre-Ora serrata	25.6 ± 6.5	−0.11	−0.50	−2.01, 1.06	0.52

*Statistical significance of the association between axial length and retinal pigment epithelium cell density.

RPE cell density was significantly lower in the SHM-group than in the remaining group for the measurements obtained at the posterior pole (*P* = 0.01), the midpoint posterior pole/equator (*P* = 0.02), and at the equator (*P* < 0.001), while the difference close to the ora serrata was not significant (*P* = 0.09) (Table 1). If the SHM-group was compared with the PHM-group, the difference in the RPE cell density was significant for the measurements obtained at the posterior pole (*P* = 0.009), midpoint posterior pole/equator (*P* = 0.04), and equator (*P* = 0.001), while the difference was not statistically significant for the measurements made close to the ora serrata (*P* = 0.11) (Table 1). If the PHM-group and the NHM-group were compared with each other, the difference in RPE cell density was significant for the measurement obtained at the midpoint posterior pole/equator (*P* = 0.035) and at the equator (*P* < 0.001), while the readings made at the posterior pole (*P* = 0.35) and close to the ora serrata (*P* = 0.52) were not significant (Table 1).

**Figure 4:**
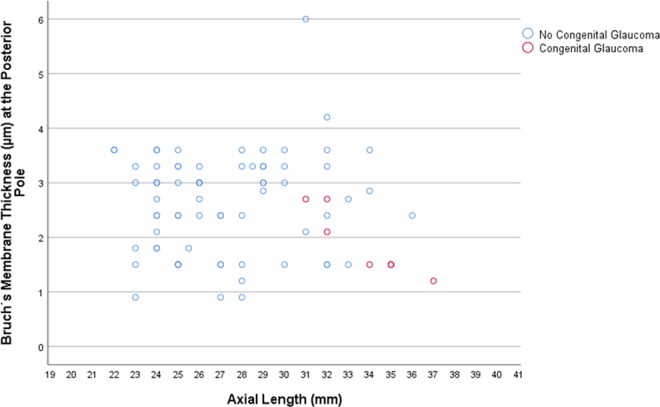
Scatterplot showing the association between axial length and Bruch’s membrane thickness at the posterior pole in eyes with congenital glaucoma versus eyes without congenital glaucoma.

BM thickness measurements were available for six eyes with congenital glaucoma and 72 eyes without congenital glaucoma. In the SHM group, BM thickness decreased with longer axial length at the posterior pole (*P* = 0.003) (Fig. 4), at the midpoint posterior pole/equator (*P* = 0.035) and at the equator (*P* = 0.017), while close to the ora serrata the measurements of the BM thickness were not associated with axial length (*P* = 0.83) (Table 2). In the PHM group and the NHM group, all BM thickness measurements were not statistically significantly correlated with axial length (Table 2) (Fig. 4).

**Table 2:** Results of the linear regression analysis of the association between the thickness of Bruch’s membrane (µm) and axial length.

Region	Bruch’s membrane thickness (µm)	Standardized Regression Coefficient beta	Non- Standardized Regression Coefficient B	95% Confidence Interval of B	*P*-Value*
**Secondary High Myopia (Congenital Glaucoma)**					
Posterior pole	1.9 ± 0.6	−0.93	−0.27	−0.39, −0.14	0.003
Midpoint posterior pole and equator	1.7 ± 0.6	−0.79	−0.22	−0.42, −0.02	0.035
Equator	1.8 ± 0.5	−0.84	−0.21	−0.37, −0.06	0.017
Pre-Ora serrata	1.7 ± 0.7	−0.10	−0.03	−0.42, 0.35	0.83
**No Secondary High Myopia (No Congenital Glaucoma)**					
Posterior pole	2.6 ± 0.9	0.11	0.01	−0.01, 0.03	0.37
Midpoint posterior pole and equator	2.6 ± 1.0	0.08	0.02	−0.05, 0.10	0.51
Equator	2.5 ± 1.0	0.21	0.06	−0.01, 0.13	0.09
Pre-Ora serrata	2.6 ± 1.2	−0.09	−0.03	−0.12, 0.05	0.45
**Primary High Myopia**					
Posterior pole	2.7 ± 1.0	0.13	0.05	−0.07, 0.17	0.43
Midpoint posterior pole and equator	2.6 ± 1.0	0.02	0.01	−0.12, 0.13	0.91
Equator	2.7 ± 1.1	0.10	0.04	−0.09, 0.17	0.54
Pre-Ora serrata	2.6 ± 1.0	−0.12	−0.05	−0.18, 0.08	0.45
**Non-Highly Myopic Group**					
Posterior pole	2.5 ± 0.8	−0.21	−0.18	−0.52, 0.15	0.28
Midpoint posterior pole and equator	2.5 ± 1.0	0.03	0.04	−0.39, 0.46	0.87
Equator	2.3 ± 0.7	0.10	0.08	−0.24. 0.41	0.60
Pre-Ora serrata	2.7 ± 1.4	−0.09	−0.14	−0.75, 0.48	0.65

*Statistical significance of the association between axial length and Bruch’s membrane thickness.

BM thickness was significantly lower in the SHM-group than in the PHM-group at the posterior pole (*P* = 0.01), at the midpoint posterior pole/equator (*P* = 0.007), at the equator (*P* = 0.006), and close to the ora serrata (*P* = 0.02) (Table 2). BM thickness did not differ significantly between the PHM-group and the NHM-group (all *P* > 0.10) (Table 2).

**Figure 5:**
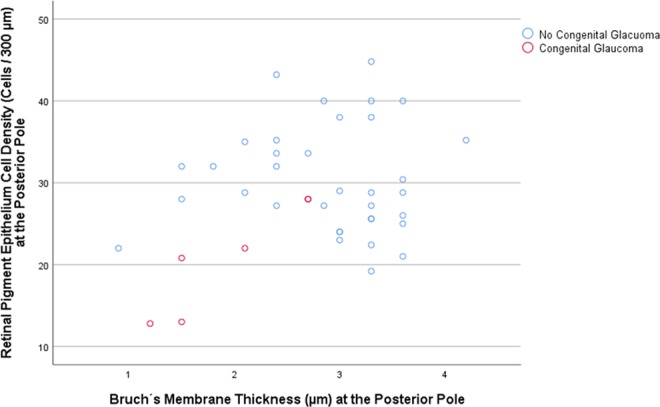
Scatterplot showing the association between Bruch’s membrane thickness and retinal pigment epithelium cell density at the posterior pole in eyes with congenital glaucoma versus eyes without congenital glaucoma.

In the SHM-group, the RPE cell density decreased with a lower BM thickness (beta: 0.93; B: 0.09; 95% CI: 0.04, 0.14; *P* = 0.007), while in the eyes without congenital glaucoma the relationship was not statistically significant (*P* = 0.98) (Fig. 5).

## Discussion

In our histomorphometric study, SHM was associated with a decreasing RPE cell density and thinning of BM with longer axial length, while in PHM and NHM BM thickness in any location and the RPE cell density at the posterior pole and close to the ora serrata were not associated with axial length. Correspondingly, BM was thinner and the RPE cell density was lower in the SHG group than in the PHM-group and the NHM-group. In the same manner, the RPE cell density decreased with a thinner BM thickness in the SHM-group while in the eyes without congenital glaucoma RPE cell density and BM thickness were not significantly correlated with each other.

The findings of a decreasing RPE cell density and a thinning of BM with longer axial length in eyes with SHM are novel and extend the observations made in a previous investigation on Chinese eyes in which highly myopic eyes with congenital glaucoma showed a tendency towards, however not a statistically significant association of, a lower RPE cell density and of a lower BM thickness with longer axial length^6^. The significant difference in BM thickness between eyes with SHM and eyes with PHM or non-highly myopic eyes as another finding of our study agrees with a similar observation made in previous studies on mostly the same eyes. Another novel observation in our study was the relationship between a thinning of BM and a decrease in RPE cell density in the SHM-group as compared to the PHM-group and the NHM-group. The finding of BM thickness decreasing with longer axial length in the SHM-group in association with a lower BM thickness in eyes with SHM in contrast to eyes with PHM also agrees with the observation made in another investigation, in which non-highly myopic eyes and highly myopic eyes without congenital glaucoma did not differ in their BM thickness nor did they show a relationship between axial length and BM thickness^5,6^.

The observation that the RPE cell density, and correspondingly, BM thickness, decreased in the SHM-group fits with the spherical enlargement of the globe in eyes with congenital glaucoma, leading to the development of a secondary macrocornea with corneal thinning, and thinning of the sclera, choroid, retina and BM throughout the globe^10^. It agrees with the notion that in SHM the elevated intraocular pressure as the primary cause of the globe enlargement leads to a stretching and lengthening of BM and of the other ocular layers. The finding that the SHM-group differed from the PHM-group and from the NHM-group in that BM thickness was not significantly correlated with axial length in the eyes without congenital glaucoma supports the hypothesis of BM being the primary driver for the axial enlargement in eyes without congenital glaucoma. That hypothesis suggests that the axial elongation of myopic eyes occurs by the new production and enlargement of BM in the mid-peripheral region of the fundus^7^. Since according to the hypothesis the RPE cells in the midperiphery produce a larger surface area of their basal membrane (i.e. BM) while their cell number may remain unchanged, it would explain the decrease in the RPE cell density in the equatorial and retro-equatorial region while the RPE cell density at the posterior pole is primarily not affected. Subsequently, BM thickness is not decreased at any location of the eye, and the thickness of the retina and the density of the RPE layer are reduced only in the mid-periphery of the fundus, while the thickness of the retina and BM and the density of the RPE cells in the macular region are not related to axial length^3,5,8,9^. It may also explain that the best corrected visual acuity is independent of the axial length in myopic eyes as long as eyes with a myopic maculopathy are excluded^12^.

The notion of BM production in the fundus midperiphery as the driver of the axial elongation could also explain the development and enlargement of parapapillary gamma zone. The optic nerve head can be regarded as a three-layered hole in the coats of the globe, with BM opening forming the inner layer, the choroidal opening the middle layer, and the scleral opening covered by the lamina cribrosa forming the outer layer. A recent optical coherence tomographical study showed that in medium myopic eyes the three layers often are not aligned to each other but that medium myopic eyes often show an overhanging of BM into the intrapapillary region on the nasal side of the optic disc^13^. Correspondingly, there is a lack of BM at the temporal optic disc side what is the equivalent of parapapillary gamma zone (as defined by the absence of BM). This backward shift of BM opening in medium myopic eyes might be explained by the production of BM in the fundus midperiphery pushing the BM in the posterior region, including BM opening, backwards, while the sclera with its opening is only passively moved and thus has the tendency to stay behind. Interestingly, BM opening area did not increase with longer axial length in eyes with an axial length of less than 26.5 mm, while beyond that limit, BM opening enlarged. It could explain the development of a circular peripapillary gamma zone with gamma zone present also at the nasal optic disc side in highly myopic eyes with an axial length of more than 26.5 mm.

Differences between eyes with SHM and eyes with PHM or non-highly myopic eyes are thus the spherical globe enlargement of the SHM-eyes versus the axially elongation of the myopic eyes without congenital glaucoma, and the involvement of all ocular regions in SHM-eyes versus the primary involvement of the equatorial and retro-equatorial region in myopic eyes without congenital glaucoma. The specific involvement of the equatorial and retro-equatorial region in the process of axial elongation in myopic eyes without congenital glaucoma would explain the decrease in the RPE cell density with longer axial length in that region as it was also found in the eyes of the present investigation (Figs. 2 and 3). It would also explain that the RPE cell density at the ora serrata and at the posterior pole were not correlated with axial length, not even in extremely myopic eyes with an axial length of more than 30 mm, as also was observed in the globes included into the present study (Table 1) (Fig. 1)^5,6,8,9^.

When discussing the findings obtained in our study, we also have to consider its limitations. First, the dimensions of the enucleated eyes were affected by the process of fixation and to a minor degree by a post-enucleation swelling before the fixative agent got effective. These preparation-associated changes in the macroscopical and microscopical measurements of the ocular tissues and layers may have affected the globes to the same degree, so that differences between the SHM-group, the PHM-group and the NHM-group might not have been influenced by that limitation of the study. In addition, it may be unlikely, that the thickness of BM as an avascular and acellular layer and the RPE cell density might at all have been affected by the preparation of the globes. Second, due to its design as a retrospective histologic study, our investigation had a selection bias. In particular, the eyes included into our study were enucleated for specific clinical reasons, so that the results obtained may not directly be generalizable to eyes without these diseases. In view of the scarcity of enucleated human globes, however, there may not be a major alternative to avoid this limitation. Third, serial sections of the globes were not available so that it could not be confirmed that the section was located exactly in center of the optic nerve head.

In conclusion, in eyes with SHM in contrast to eyes with PHM or non-highly myopic eyes, the RPE cell density, and in a parallel manner BM thickness, decrease with longer axial length. The findings fit with the notion of BM being a primary driver in the process of axial elongation in PHM as compared to SHM.
